# Insights into Synthesis and Optimization Features of Reverse Osmosis Membrane Using Machine Learning

**DOI:** 10.3390/ma18040840

**Published:** 2025-02-14

**Authors:** Weimin Gao, Guang Wang, Junguo Li, Huirong Li, Lipei Ren, Yichao Wang, Lingxue Kong

**Affiliations:** 1 School of Metallurgy and Energy, North China University of Science and Technology, Tangshan 063600, China; weimin.gao@outlook.com (W.G.); lijg99@163.com (J.L.); lihuirong@ncst.edu.cn (H.L.); 2 Institute of High Energy Physics, Chinese Academy of Sciences, Beijing 100049, China; wangguang@ihep.ac.cn; 3 Institute for Frontier Materials, Deakin University, Locked Bag 20000, Geelong, VIC 3220, Australia; renlip@deakin.edu.au; 4 School of Science, RMIT University, Melbourne, VIC 3000, Australia

**Keywords:** feature identification, synthesis, machine learning, reverse osmosis, membrane performance

## Abstract

Reverse osmosis membranes have been predominantly made from aromatic polyamide composite thin-films, although significant research efforts have been dedicated to discovering new materials and synthesis technologies to enhance the water–salt selectivity of membranes in the past decades. The lack of significant breakthroughs is partly attributed to the limited comprehensive understanding of the relationships between membrane features and their performance. Insights into the intrinsic features of reverse osmosis (RO) membranes based on metadata were obtained using explainable artificial intelligence to understand the relationships and unify the research efforts. The features related to the chemistry, membrane structure, modification methods, and membrane performance of RO membranes were derived from the dataset of more than 1000 RO membranes. Seven machine learning (ML) models were constructed to evaluate the membrane performances, and their applicability for the tasks was assessed using the metadata. The contribution of the features to RO performance was analyzed, and the ranking of their importance was revealed. This work holds promise for metadata analysis, evaluating the RO membrane against the state of the art and developing an inverse design strategy for the discovery of high-performance RO membranes.

## 1. Introduction

Reverse osmosis (RO) membranes are widely used in seawater desalination to address the current global water crisis [1]. They are semi-permeable membranes that allow pure water to flow from the side of higher solute concentration to the other side of lower solute concentration under applied pressure. Following the successful introduction of cellulose acetate (CA) RO membranes [2], the interfacial composite polyamide membrane was developed in 1972 [3], which featured a thin-film composite design, with better rejection and flux at lower operating pressures than the cellulose acetate membranes. Ultimately, the polyamide-based materials became the dominant chemistry for the thin-film composite (TFC) membranes used in RO processes today, due to their higher rejection, higher flux, higher strength, and lower energy consumption. Compared to the CA membrane, the salt rejection and water flux of a polyamide (TFC) membrane is increased from 85–95% to 96–99.8% and from 5–30 Lm^−2^h^−1^ to 10–75 Lm^−2^h^−1^, respectively, and the energy consumption is reduced by about 30% for seawater desalination. The RO membranes are typically composed of multiple layers. The common structures of RO membranes include cellulose acetate membranes, asymmetric membranes, thin-film composite membranes, thin-film nanocomposite membranes, and hybrid membranes. In this study, we will focus on the last four types of membranes. Their structures can be characterized by a thin, dense selective surface layer on top of a porous support sublayer, and they will be referred to as porous RO membranes in this work.

Nevertheless, TFC polyamide membranes exhibit fouling tendencies [4,5], susceptibility to chlorine degradation [6], and a trade-off between permeability and selectivity [7,8]. In recent decades, a significant effort has been focused on improving this thin-film membrane design by developing new core chemistries for the selective layer and optimizing the processing or manufacturing to improve membrane performance and break the limitations of polyamides. As a result, a wide variety of chemistries have been explored, and numerous desalination membranes for either seawater or brackish water have emerged in recent years. Brackish water RO (BWRO) applications are generally under low applied pressures (below 20 bar) and low feed salinity (2000 mg/L), whereas the operating conditions of seawater RO (SWRO) membranes are much harsher (≥32,000 mg/L feed salinity with applied pressure over 50 bar). Standard testing conditions for seawater and brackish water RO processes were introduced based on industry-reported conditions [9]. There are many articles that have reviewed the significant number of research works on TFC membranes [10,11,12,13,14], highlighting the prospects and challenges of the next breakthrough in desalination and water purification membranes.

Following the accumulation of experimental, computational simulation, and commercial product data on membranes, machine learning has been applied in membrane design and evaluation [15,16,17], inspired by its success in materials discovery [18]. The application includes membrane design [19], membrane property prediction [20], gaining insights into property–performance relationships [21], modeling membrane fouling and performance [22,23,24,25], and optimizing system performance [26,27,28].

Various machine learning algorithms have been used to formulate prediction models, including linear and decision tree regression, artificial neural networks (ANNs), ensemble learning models, such as Random Forest (RF), hybrid models combining ML with physical or empirical models, and deep learning (DL) for time-series data or complex water chemistry datasets. The ML prediction models allow for better system design, real-time monitoring, and operational adjustments. By using ML, the rapid virtual screening and optimization of membranes become possible. To advance further, ML models offer significant potential to accelerate the design and discovery of membrane materials and support the inverse PSPP (Processing–Structure–Properties–Performance) design of processes tailored to the desired target performance [29,30]. However, there are also challenges in implementing these advanced applications, such as ensuring the accuracy and reliability of the models when dealing with diverse and complex real-world data, which need to be addressed for the full realization of this potential.

This work is centered on screening membrane synthesis and manufacturing techniques, with the goal of advancing membrane development. Data from the Open Membrane Database (OMD), available at www.openmembranedatabase.org, were utilized. The data were heterogeneous and provided a limited representation of the actual long-term operating conditions of membranes in industrial applications, but they remain a valuable resource containing comprehensive information on the various aspects of membranes at present, providing a solid foundation for this study. Seven ML models were formulated to predict the ratio of water permeability and salt selectivity of RO membranes from the OMD. The predictive capabilities of these models were evaluated using the root mean square error (RMSE), the mean absolute error (MAE), and the R-squared (R^2^) metrics. Furthermore, SHAP (SHapley Additive exPlanations), an explainable AI (XAI) approach, was applied to enhance the interpretability of these models. This approach was crucial as it identified which features had the most significant impact on the membrane performance predictions, enabling more informed decisions in the membrane development process. Specifically, for the purpose, this work focused on the chemistry, membrane structure, and modification methods of RO membranes. The contribution and importance of these features to membrane performance were revealed using SHAP coefficients, making the predictions more transparent and actionable.

## 2. Methodology

### 2.1. Dataset and Data Processing

The datasets used in this study were obtained from the Open Membrane Database (OMD) [9]. A total of 1158 desalination membranes were collected from peer-reviewed journals, patents, and commercial product datasheets. OMD is a well-curated repository that compiles data from these reliable sources, ensuring the quality and relevance of the information. Further information on the database can be found at www.openmembranedatabase.org. The datasets included detailed information such as the reporting source, experimental conditions, chemistry, synthesis modification, physical characteristics, and performance of each membrane. As the data came from various sources, both the measurement methods and reporting standards were variable. It was challenging to integrate and compare the datasets from different studies, as well as provide consistency performance evaluations. Standard testing conditions were defined in the OMD for seawater and brackish water RO processes. In the datasets, the water permeability coefficient, A (L m^−2^ h^−1^ bar^−1^), and the salt permeability coefficient, B (L m^−2^ h^−1^), were provided. The former was calculated by normalizing the permeate water flux across the membrane, J_w_, by the difference between the applied hydraulic pressure, ΔP, and the osmotic pressure, Δπm. The latter was determined from water flux, J_w_, and salt rejection, R_j_. This calculation did not take into account the concentration polarization, although the observed salt rejection could be calculated using the salt concentrations in the feed and permeate. This study considered the water–salt selectivity (i.e., the ratio of the water and salt permeability coefficients, A/B) as the performance characteristic of membranes. The selection of water–salt selectivity as the performance characteristic is justified because it provides a comprehensive measure that integrates both the ability of the membrane to allow water to pass through and its effectiveness in rejecting salt, offering a holistic view of the membrane performance in desalination processes. The use of A/B is essential [14,31]. An upper-bound could be clearly illustrated when A/B was used as the *y*-axis parameter. The upper bound in A/B against A made the evaluation of the TFC membranes simpler without the need to ascertain the height of the PA active layer. A trade-off relationship between permeability and selectivity could be well established in the form of the upper bound.

The intrinsic membrane features were derived from the datasets and, along with the filtration mode and the concentration polarization (CP) modulus, were considered as the input variables in modeling. The predominant materials of the selective layer were the primary features considered in the model. The structures of the selective layer included asymmetric, TFC, thin-film nanocomposite (TFN), and inorganic as described in OMD [9]. Membrane modification was classified into the following two categories: (1) optimization of the selective layer by finetuning the synthesis conditions, such as the use of additives and monomers and applying deposition, and (2) post-treatment modification after synthesis, such as surface activation to increase hydrophilicity or improve antifouling properties, annealing to optimize pore structure or enhance polymer cross-linking, and solvent treatment to rearrange polymer chains, remove residual monomers, or improve adhesion between the polyamide selective layer and the porous support layer. The optimization of the support and selective layers and the various modification methods for different improvement strategies can be found in the literature [14].

The inclusion of CP in the model inputs was necessary because the published values of A and B in the dataset were derived from reported membranes without considering the influence of CP. Since the concentration of ions at the membrane surface on the feed side differs from that in the bulk solution due to CP, ignoring this effect could influence the prediction accuracy.

To account for the effect of CP on A and B, the ion concentration at the membrane surface can be approximated by using the boundary layer film model, which is based on the principle of mass transfer across a thin layer adjacent to the membrane surface and accounts for factors such as the flow conditions and concentration gradients. The mass transfer coefficient at the CP boundary layer, *k*, depends on the hydrodynamic conditions over the membrane surface [32]. It can be calculated experimentally by conducting specific tests that measure the mass transfer rates under controlled conditions or estimated theoretically from the dimensionless equations [33,34,35], which provide a theoretical framework for estimating *k*.

### 2.2. ML Models

Seven ML models were employed in this study. A multi-layer perceptron (MLP) was first chosen as it is a powerful tool for various machine learning tasks. It can learn complex patterns in data by using nonlinear activation functions in the neurons [36]. K-nearest neighbors (kNN) is a simple and very effective supervised machine learning algorithm. It is a type of instance-based learning, making predictions for a new instance based on the mean or the median of the K-most similar instances. Support vector machine (SVM) is also a conventional ML algorithm frequently applied for classification and regression tasks in various fields. SVM works by finding an optimal high-dimensional hyperplane to separate the data into different classes, where the nearest data points (support vectors) of each class have the maximum margin from the hyperplane [37]. Support Vector Regression (SVR) uses the same principles as SVM for predicting continuous outputs [38].

For comparison, four ensemble models, AdaBoost, CatBoost, XGBoost, and Random Forest (RF), were also applied in this study. AdaBoost combines multiple weak learners to make a strong classifier or regressor. The first estimator is built by fitting the original dataset and then a new estimator is built based on the errors of the previous model to improve the predictions [39]. Unlike AdaBoost, Catboost and XGBoost are gradient boosting algorithms that work on the principle of the stagewise addition method. In addition to handling numerical features, CatBoost can handle categorical features without preprocessing, whereas XGBoost requires preprocessing. Random Forest (RF) is a type of Bagging algorithm [40]. Although its development can be traced to 2001, it has been one of the most popular and powerful machine learning algorithms, showing performances similar to those of other tree-based models, such as XGBoost. CatBoost, XGBoost, and Random Forest were employed because they are well suited for structured tabular data and have strong feature importance capabilities.

The Bayesian optimization method was used to estimate the hyperparameters of these ML algorithms. Bayesian optimization is an efficient approach for finding the optimal hyperparameter values in large-scale problems by leveraging probabilistic models, reducing the number of required iterations compared to a grid search. The dataset was split into 80% training and 20% validation, and cross-validation was applied to ensure robustness. To prevent overfitting, we implemented early stopping, L2 regularization, and pruning strategies for tree-based models. It was found that the validation of the generated models produced approximately the same scores as the training phase. This indicates well-generalized models and well-avoided overfitting. In the preparation stage, variables with less than 10% missing data were handled using mean imputation for numerical variables and mode imputation for categorical variables. Variables with a higher percentage of missing data were filtered out. Comparing the capabilities of these general ML models and ensemble ML models was performed to understand which model would be more suitable for studying the desalination performance of the RO membranes and to identify the importance of their intrinsic features in relation to the water–salt selectivity.

### 2.3. Feature Importance Analysis

SHAP analysis was employed to gain a deeper understanding of how each feature affects the target [41]. This method is based on coalitional game theory and provides SHAP values to measure the contribution of each input feature to an individual prediction. The SHAP method can be seamlessly integrated into supervised ML models [42], including decision trees, gradient boosting models, RF, and neural networks. In this study, the SHAP analysis was executed for the models showing better performance. The results were analyzed to comprehensively elucidate the significance of the features.

## 3. Results and Discussion

### 3.1. Comparison of ML Models

**Figure 1:**
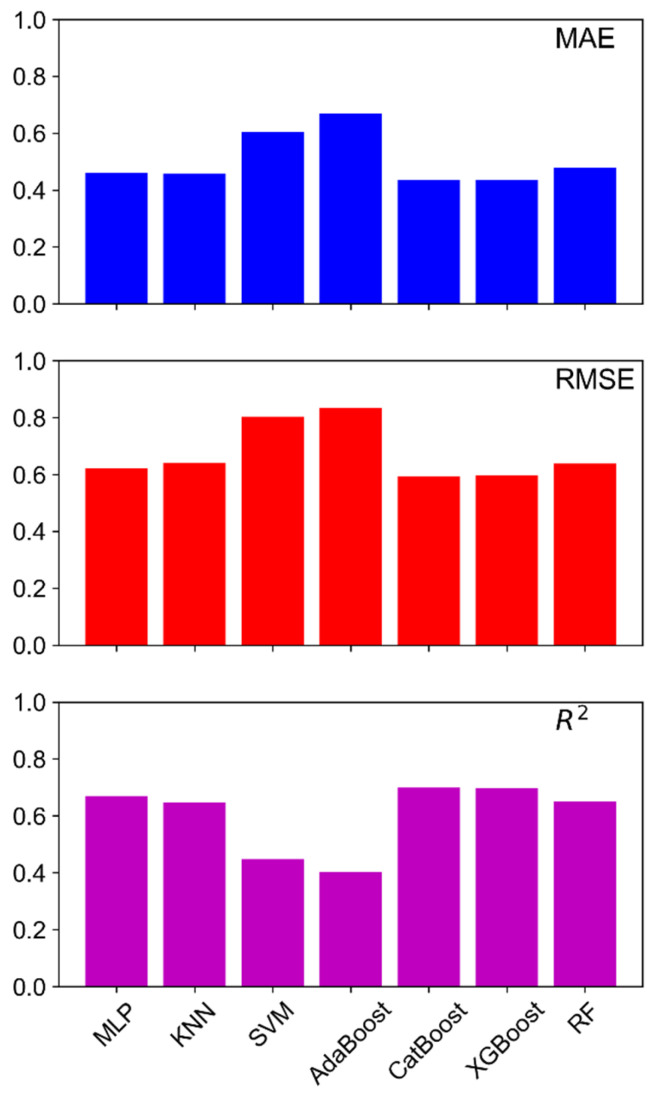
Summary of the model performance metrics of mean absolute error (MAE), root mean square error (RMSE), and R^2^ score generated by the validation datasets of the ML models.

The performance and prediction errors of each model were first evaluated with the mean absolute error (MAE), root mean square error (RMSE), and R-squared (R^2^) metrics to determine the best models, as shown in Figure 1. These metrics provide valuable insights into the model performance, quantifying the accuracy and error rates of the model predictions. To enhance the robustness of the evaluation, k-fold cross-validation (with k = 5) were employed in the present work as mentioned earlier. Cross-validation divided the dataset into multiple folds, ensuring that each data point was used for both training and validation, reducing bias and variance in performance estimates and helping to mitigate overfitting.

SVM and AdaBoost clearly showed higher MAE and RMSE and lower R^2^, exhibiting weak performance for the datasets. In particular, AdaBoost represented the worst performance among the seven ML models. The key for the algorithm is to have a diverse enough set of weak learners [39]. Despite carrying out tuning of the model to ensure that it performs at its best, the suggested estimators of 378 may not have captured enough information to achieve high effectiveness and improve the overall performance of the ensemble ML model significantly. AdaBoost typically uses shallow decision trees as weak learners, which may not capture the complex relationships between features in the high-dimensional datasets. This is also due to the model which is incapable of handling categorical data and imbalanced distributions. SVM presented the second worst performance among the ML models. The general ML models, MLP and kNN, showed the same prediction capability as the ensemble ML models, CatBoost and XGBoost. However, RF presented a slightly lower performance compared to the four models. Generally, CatBoost and XGBoost are efficient in handling nonlinear relationships, missing data, and structured tabular data. While MLP can also learn complex patterns, it requires a large dataset to generalize well. In contrast, kNN performs well in low-dimensional, well-structured datasets but struggles with scalability. The dataset used in the present work is large, with more than 1000 samples, and well structured, and any missing data were imputed to ensure completeness.

**Figure 2:**
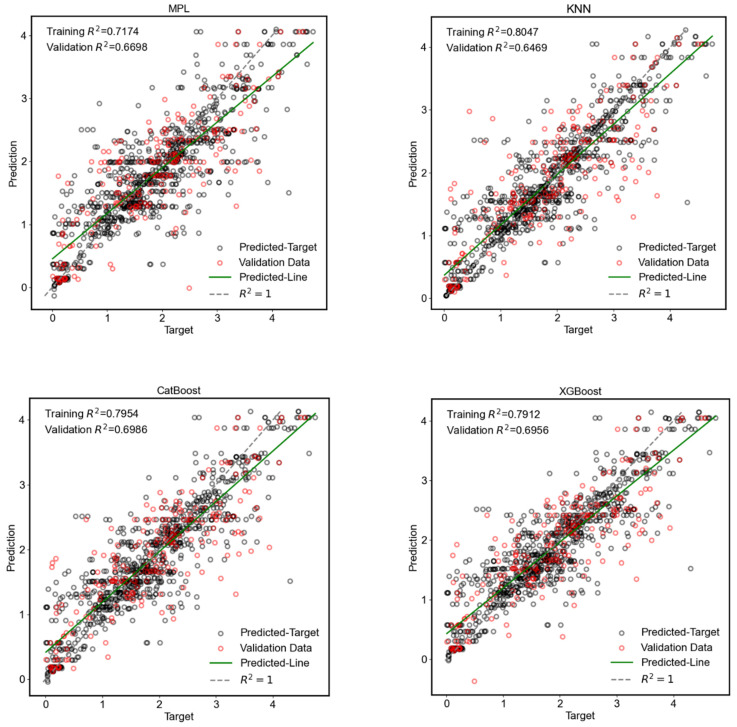

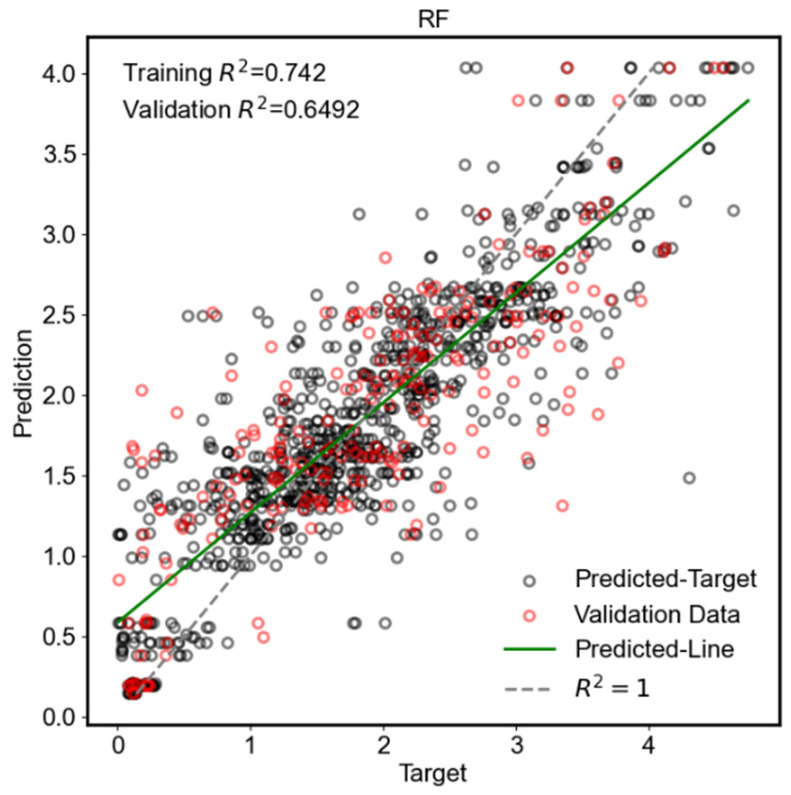
Comparison of different ML models by analyzing the predicted and targeted output and R^2^ score of each model.

Figure 2 illustrates the comparison between the target and predicted outputs from the trained ML models for the training and test datasets. The training R^2^ value provides an indication of how well the model fits the training data. The predicted line represents the tendency of the predicted values against the actual target values, and the angle and distance between the predicted line and the R^2^ = 1 line reveals the degree of overall mismatch. Together, these aspects show the performance in the training phase. The validation dataset and the corresponding predicted values using the trained model are also presented in the same figure, which allows us to observe the sampling distribution for validation. Along with the validation R^2^, the comparison between the validation samples and predicted data helps in assessing the goodness of prediction. The comparison is also essential to confirm the accuracy, reliability, and generalization ability of the models to new data.

The five models presented similar performance during both the training and validation phases, with training R^2^ values in the range of 0.7174–0.8047 and validation R^2^ values in the range of 0.6469–0.6986. The overall R^2^ values, which include the training and validation data, are 0.7072, 0.7708, 0.7746, 0.7706, and 0.7225 for MLP, kNN, CatBoost, XGBoost, and RF, respectively.

kNN shows the highest R^2^ for the training dataset but the lowest R^2^ for the validation dataset. This suggests that the kNN model is overfitting to some extent to the training data and failing to generalize well with the unseen data. The dataset includes 43 input features, and the high-dimensional nature makes kNN unreliable for generalization. CatBoost and XGBoost present approximately the same training and prediction performance, with R^2^ values of about 0.7. The performance of the MLP model for the training and validation dataset is lower, with an overall R^2^ value of 0.7072, although the hyperparameters of the model were determined by KerasTuner, which is generally capable of producing a high-quality model. A high-performance ANN network can be obtained by improving the MLP model and adapting the DL model for a given number of input and output features. As the datasets are large and high-dimensional, a deeper learning model may better capture intricate data patterns. The integration of convolutional layers (CNNs) or transformer-based architectures can be considered, as sequential relationships exist in the synthesis–structure–property–performance process of RO membrane application.

### 3.2. SHAP Analysis

**Figure 3:**
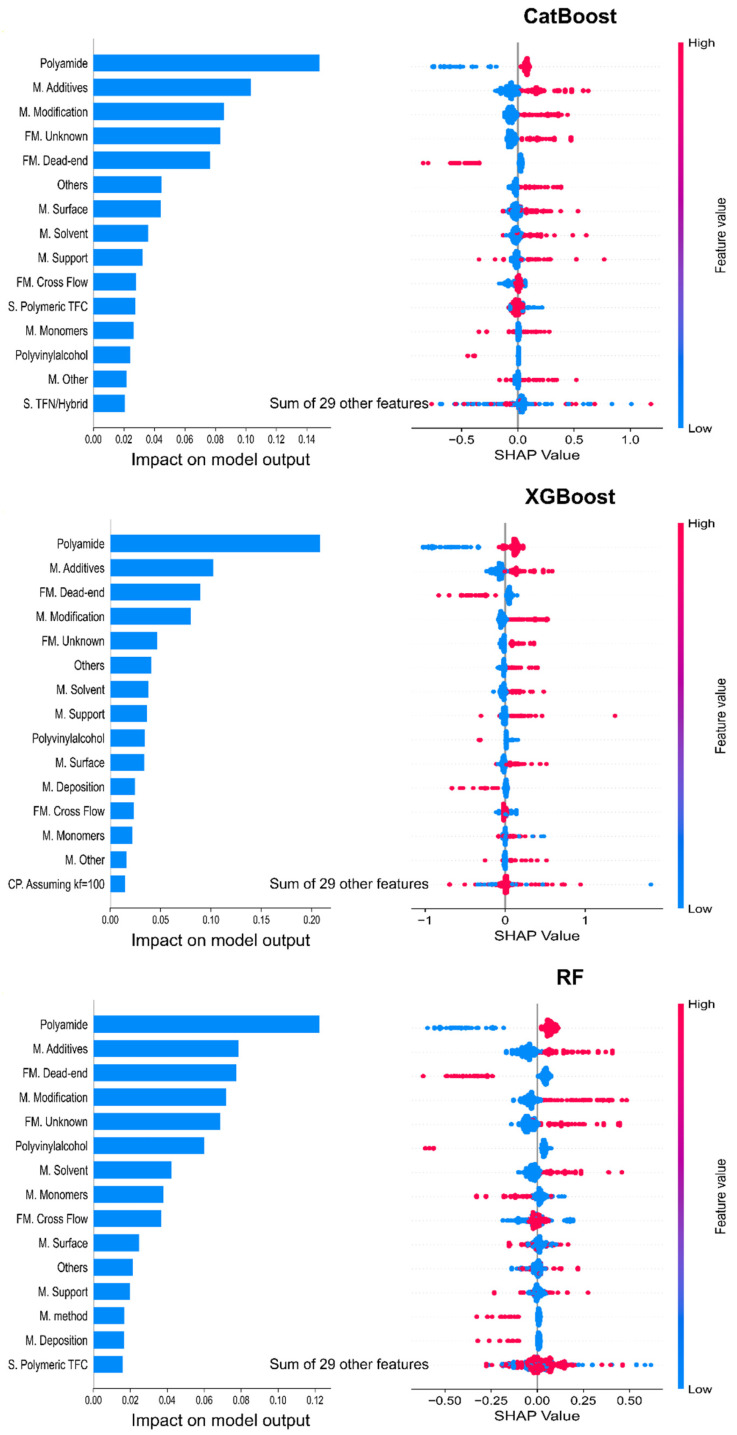
SHAP values determined from CatBoost, XGBoost, and RF for individual membrane features. (M.: Modification, FM. Filtration mode, CP.: Concentration Polarization, S. Structure).

CatBoost and XGBoost exhibited the highest R^2^ values and the lowest MAE and RMSE values among the ML models adapted in this study. The quantitative analysis revealed that they had approximately the same cross-validation score. They are indeed the most similar models. Moreover, RF showed the highest cross-validation score among all those models. Considering that XGBoost also demonstrated highly competitive performance in terms of the other evaluation metrics (R^2^, MAE, and RMSE) and its known advantages in handling various types of data and scalability, XGBoost and RF were selected as the best-performing models. SHAP analysis was performed for the three models. The following two values were estimated for each model for each input membrane feature: mean absolute SHAP value (impact on model output) and SHAP value showing the distribution of the impacts of each feature on the output. The results are shown in Figure 3. Here, only the first 15 important features are illustrated in order to focus on the most influential features that have a significant impact on the model output and to simplify the presentation and analysis without getting overwhelmed by a large number of features with relatively minor contributions. The three models produced similar results, showing a congruous rank of features with each other. A comprehensive SHAP value analysis and feature ranks are discussed with significance results in the following section.

SHAP quantified the contribution of individual features to the predicted A/B based on the training and test datasets. However, the results depend on the quality of the input data. If the dataset contains biases, missing values, noise, or highly correlated features, the SHAP explanations may be misleading, making it difficult to determine the true impact of individual variables. As mentioned earlier, the OMD dataset is heterogeneous and lacks completeness in some membrane properties and cost-related data. While these dataset characteristics may influence the estimated contribution of individual features to some extent, they do not compromise the reliability of the overall feature importance evaluation, as the outliers were removed and sufficient data were used for training and model validation.

### 3.3. Contribution of Features

The intrinsic membrane features considered in this study fall into the following four classes: chemistry of the selective layer, synthesis and subsequent modifications, and structure. The SHAP analysis results of the three selected models show that the most important feature is the polyamide. Its SHAP values are about 1.6 times that of the second significant feature. This indicates that the water–salt selectivity of membranes synthesized with polyamide is significantly different from that of the other materials. Insights into the dataset indicate that the mean water–salt selectivity of the polyamide membranes is 4.6 times that of other membranes. Geoffrey M. Geise [43] summarized the characteristics of polyamide reverse osmosis membranes, which have continuously improved in processing or manufacturing modifications during the past four decades. Inhomogeneities in the thickness and density of the polyamide membranes promote water transport through them.

In addition, another emphasized selective layer material is polyvinylalcohol (PVA), which also shows an observable impact on the model output. On the contrary, the PVA membranes have lower water–salt selectivity (17%) compared to the non-PVA membranes. However, they possess other advantageous properties that make them worthy of attention. For instance, PVA is known for its good hydrophilicity, which can enhance the membrane fouling resistance to some extent. Hence, although PVA-based membranes find diverse applications, the use of different PVA-based membranes for water treatment requires further investigations to develop their performance in terms of rejection, hydrophilicity, antifouling, and chemical and mechanical resistance [9]. This would expand their potential use and improve their competitiveness in the water treatment market.

**Figure 4:**
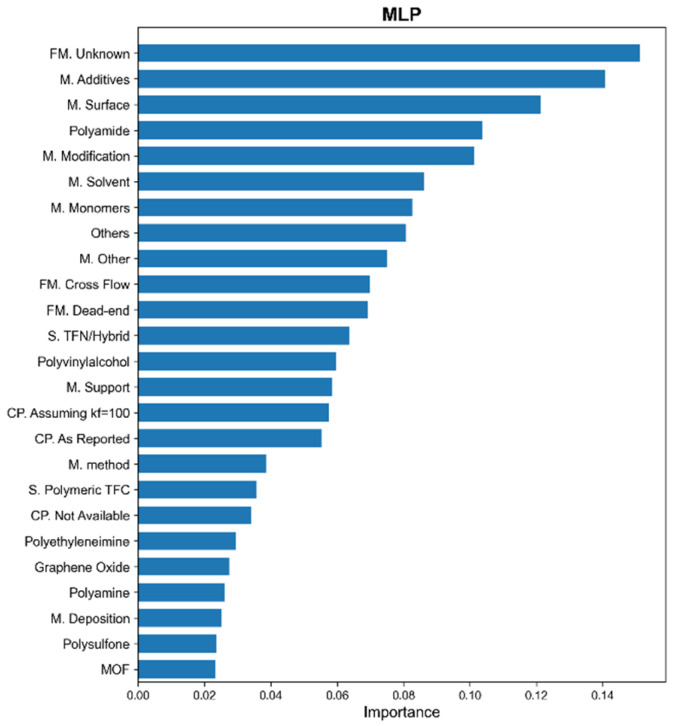
Importance from MLP for the first 25 features.

Besides the two membrane materials, modification technologies are of the utmost importance among the factors influencing membrane features. Half of the first 15 features have a large impact on the model output associated with the modification methods, e.g., 7 features based on the CatBoost and 8 features based on the XGBoost and RF models. Figure 4 shows the feature importance analysis results based on the MLP model. The results are consistent with the ensemble ML models, with a slight difference in the feature ranks. The MLP model attributes higher importance to the modification-related features, such as M. Additives and M. Surface, which are ranked higher than polyamides. The use of additives during synthesis can alter the chemical composition and functionality of the membrane. Similarly, surface treatment techniques affect membrane interaction with water and solutes. Their prominence among the important features highlights the crucial role that these modifications play in determining the overall performance of the membranes. Overall, the modification methods are the most influential features related to membrane performance.

The SHAP values show that most modification technologies are effective, whereas some, such as monomer modification and deposition techniques, are ambiguous. The application of these modifications was originally reported to be effective; however, their impact in this study appears less conclusive. The deposition of certain substances onto the active layer can significantly enhance membrane performance by improving properties such as water permeability, salt rejection, fouling resistance, and chemical stability [14]. For instance, substances such as polyethylene glycol (PEG), zwitterionic polymers, graphene oxide (GO), and polydopamine (PDA) can increase surface hydrophilicity, reducing the fouling, minimizing membrane clogging, and improving water flux without sacrificing rejection. However, the XGBoost and RF models have indicated that this kind of modification negatively influences the A/B of the RO membranes. The use of monomers during interfacial polymerization plays a key role in optimizing RO membrane performance. Modification with specific monomers during synthesis allows for the tailoring of the molecular structure of the membrane to achieve the desired performance characteristics. For example, hydroxyl-functionalized monomers can increase water transport pathways and enhance both water permeability and selectivity [14]. The CatBoost and XGBoost models produced consistent results for most samples, whereas the RF model showed inconsistent results, suggesting that this modification reduced the membrane performance.

The extensive explanation based on ML models and feature importance analysis are different for the different classes of membranes, even showing negative impacts on the output under some conditions. A comprehensive understanding of feature impacts from the ML models depends, to some extent, on the meta-information collected in the datasets. More metadata and a sufficient number of intrinsic features of individual membranes are significant for forward synthesis–structure–performance modeling. Sufficient intrinsic features allow for a more detailed representation of the membranes in the model, enabling more accurate predictions and a better understanding of how changes in these features can lead to variations in membrane performance, which is crucial for advancing membrane development.

## 4. Conclusions

Seven ML models were formulated and implemented to predict the reverse osmosis membrane performance in terms of water–salt selectivity in the form of forward PSPP relationships. The results suggest that a highly accurate conventional ANN or ensemble ML model may not be built based on the existing synthesizing and structural features, although CatBoost and XGBoost exhibited the most gratifying performances. More consistent and standardized data collection across different sources could improve the robustness of the ML models. Understanding data types and preprocessing metadata are as important as selecting the appropriate models to build accurate and transferable AI models, as the data tend to be particularly heterogeneous in terms of their type and source. By incorporating a chemical synthesis mechanism, theoretical and physical modeling, and membrane transport equations into ML models, physics-informed ML approaches may enable models to better account for input variables, leading to more accurate and reliable predictions even with limited data.

The ML models helped to screen the membrane synthesis and modification methodologies, determining whether the feature or modification was desirable for specified target membrane performance. SHAP analysis of the intrinsic membrane features was conducted using the better ensemble ML models. These models produced consistent results. Unsurprisingly, the analysis showed that polyamide-based RO membranes had the most importance, whereas polyvinylalcohol emerged as the most prominent core chemistry affecting water–salt selectivity. Among the membrane features, the modification technologies are critical in RO membrane performance improvement. The key features were revealed by cross-checking their importance from the better models, CatBoost, XGBoost, RF, and MLP. Most modification technologies are effective, whereas some show ambiguous impacts on the output. These findings suggest that the impact of some modification techniques on overall RO membrane performance may be more complex than previously reported. Further research is needed to explore the long-term stability and scalability of these modifications in industrial applications.

The insights from the SHAP analysis provide a data-driven framework for predicting how selective layer chemistry and modification impact membrane performance. With future improvements in database standardization, including harmonized testing protocols, the integration of long-term industrial data, and the expansion of reported membrane properties for practical applications, the predictive accuracy of the ML models will be improved, and this SHAP analysis can be used to design next-generation membranes with tailored properties. This work holds promise for metadata analysis, assessing the RO membrane against the state of the art and developing an inverse design strategy for high-performance RO membrane discovery.

## Data Availability

The original contributions presented in this study are included in the article. Further inquiries can be directed to the corresponding authors.
